# Supplementary material for: Mutant IDH1 Depletion Downregulates Integrins and Impairs Chondrosarcoma Growth
Source: Cancers (Basel). 2020 Jan 6;12(1):141. doi: 10.3390/cancers12010141 (PMC7017040; doi:10.3390/cancers12010141)
Supplement: Supplementary file 1 [file cancers-12-00141-s001.pdf]

Supplemental Materials

Mutant IDH1 Depletion Downregulates Integrins and Impairs Chondrosarcoma Growth

Luyuan Li, Xiaoyu Hu, Josiane E. Eid, Andrew E. Rosenberg, Breelyn A. Wilky, Yuguang Ban, Xiaodian Sun, Karina Galoian, Joanna DeSalvo, Jinbo Yue, Xi Steven Chen, Marzenna Blonska and Jonathan C.Trent

Chemical treatment

Treatment of cells with cell-permeable D-2HG was carried out by adding octyl-D-2HG (Millipore Sigma) to the culture medium to a final concentration of 100 μM, for 12 h.

Immunohistochemistry (IHC)

Immunohistochemical analysis was performed on 4-μm fine sections cut from formalin-fixed, paraffin-wax-embedded samples utilizing an antibody against ERG following a standard protocol at UMH Immunohistochemistry Laboratory. Images were obtained using VS120 Virtual Slide Microscope (Olympus) at 20× magnification.

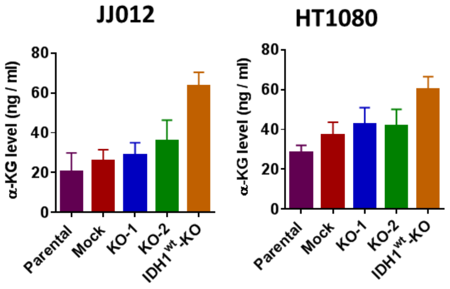

Figure S1. α-KG levels measured by HPLC-MS in the parental, mock, KO and IDH1^{wt}-transduced KO cells of JJ012 and HT1080 cell lines.

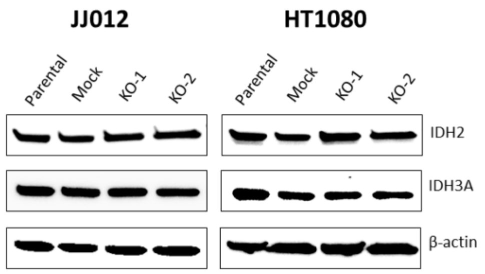

Figure S2. Immunoblot shows knockout of IDH1^{mut} does not affect the expression of IDH2 and IDH3 in JJ012 and HT1080 cells.

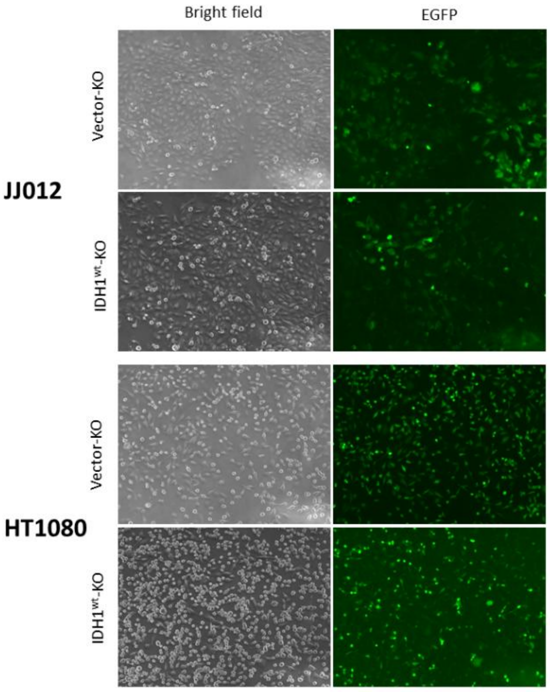

Figure S3. Overexpression of IDH1^{wt} in chondrosarcoma IDH1 KO cells. EGFP images show efficient infection of the blank (vector) and the IDH1^{wt}-expressing (IDH1^{wt}) lentiviral vectors in the JJ012 and HT1080 IDH1 KO cells.

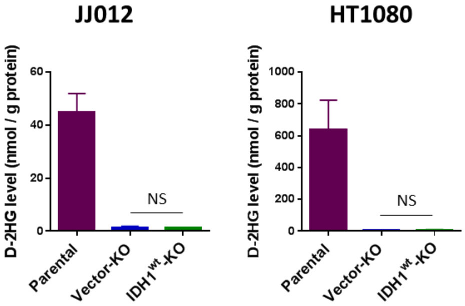

Figure S4. HPLC-MS analysis shows that re-expression of IDH1^{wt} does not alter the reduced D-2HG levels in JJ012 and HT1080 IDH1 KO cells as compared to their parental controls.

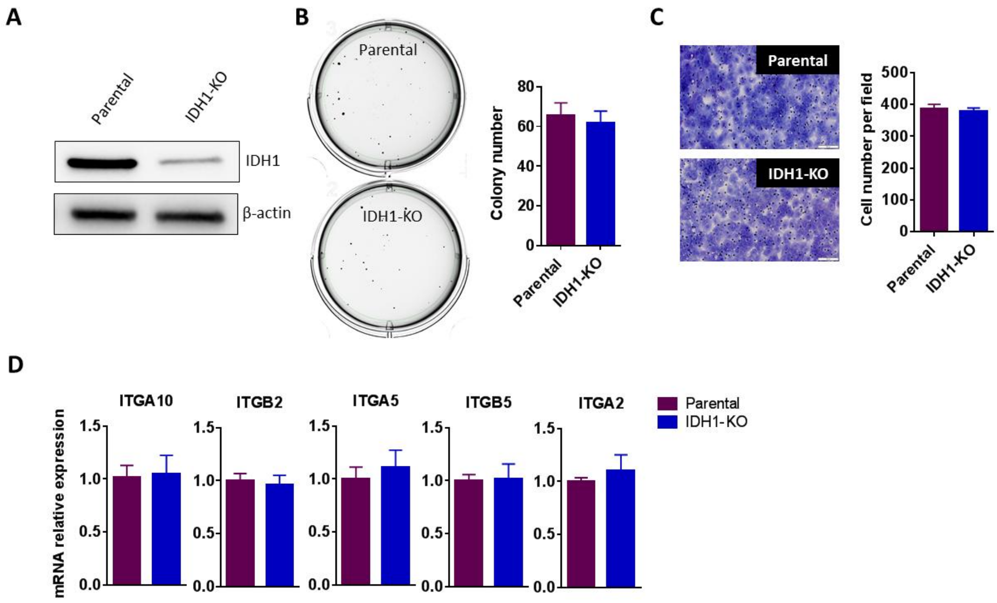

Figure S5. Knockout of IDH1 does not alter the properties of C28 chondrocytes. (**A**) Immunoblot showing significant suppression of IDH1 upon CRISPR/Cas9 knockout in the C28 chondrocytes. Loss of IDH1 does not effect a change in C28 cell capacity for colony formation in soft-agar (**B**), migration (**C**), and expression of integrins (**D**). Size bars in white represent a 100 µm scale. Cells were transfected with IDH1 CRISPR/Cas9 KO plasmids and HDR plasmids, and further selected by puromycin. A pool of IDH1-knockout cells were used for these experiments.

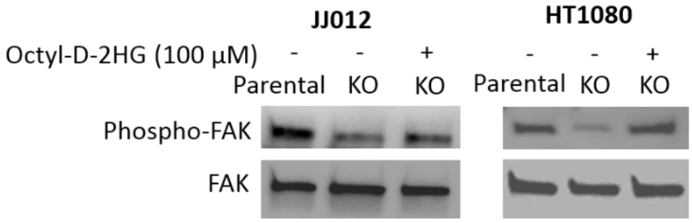

Figure S6. FAK phosphorylation at tyrosine 397 was decreased in both JJ012 and HT1080 IDH1^{mut} KO cells. Treatment with octyl-D-2HG, a membrane-permeant precursor form of D-2HG, led to a significant increase of FAK phosphorylation in these KO cells.

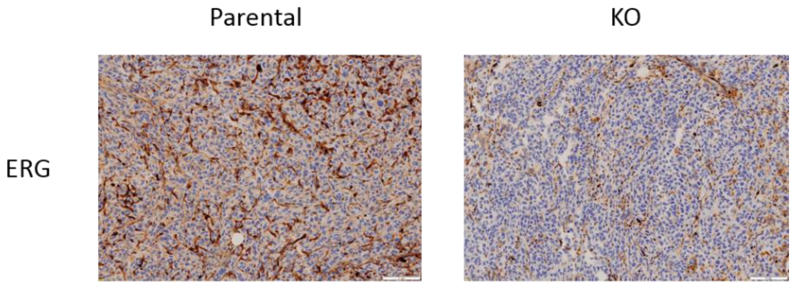

Figure S7. Expression of the endothelial marker ERG, in JJ012 parental and IDH1^{mut} KO cell derived tumors. Images were obtained using a VS120 Virtual Slide Microscope at 20x magnification. Size bars in white represent a 100 µm scale.

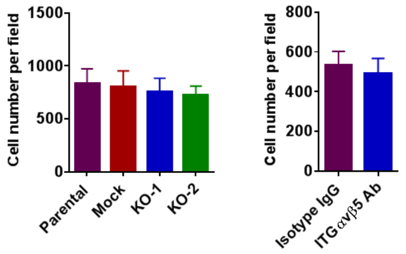

Figure S8. Vitronectin adhesion assay of indicated HT1080 groups (left) and HT1080 cells pretreated with 10 μg/ml ITGαvβ5-blocking antibody or its corresponding isotype control antibody (right). Cells were incubated on vitronectin-coated plates for 2h followed by washing, fixing, and staining. Cell adhesion was evaluated by counting the average number of attached cells per field. Results are shown as the mean ± SEM of triplicate cultures and are representative of 3 independent experiments.

Figure 1B

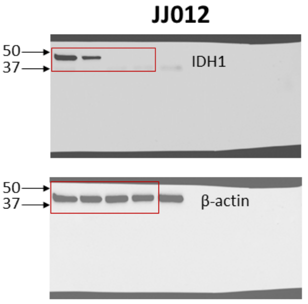

Sample	Parental	Mock	KO-1	KO-2
Density Ratio	0.85	0.45	0	0

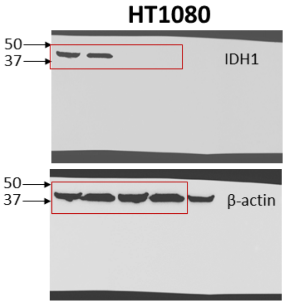

Sample	Parental	Mock	KO-1	KO-2
Density Ratio	0.86	0.62	0	0

Figure 3A

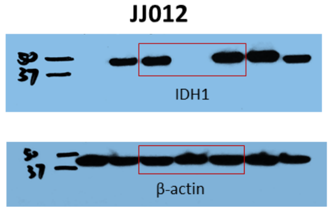

Sample	Parental	Vector-KO	$IDH1^{wt}$-KO
Density Ratio	0.97	0	1.05

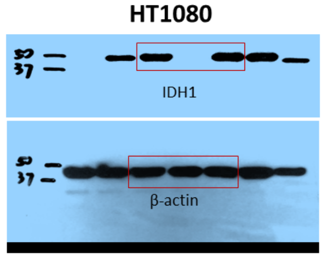

Sample	Parental	Vector-KO	$IDH1^{wt}$-KO
Density Ratio	1.03	0	1.37

Figure 4C

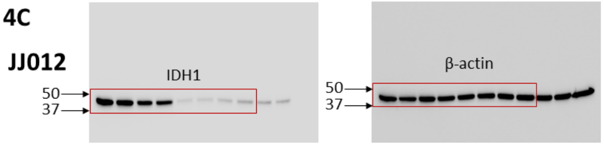

Sample	Parental	Parental	Mock	Mock	KO-1	KO-1	KO-2	KO-2
Density Ratio	1.26	1.37	0.89	0.93	0.06	0.06	0.09	0.13

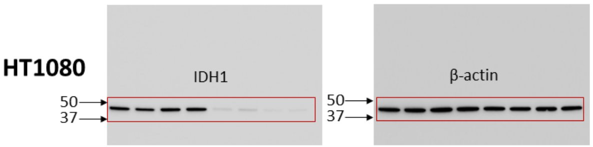

Sample	Parental	Parental	Mock	Mock	KO-1	KO-1	KO-2	KO-2
Density Ratio	1.06	0.77	0.90	0.99	0.07	0.10	0.03	0.03

Figure 5E

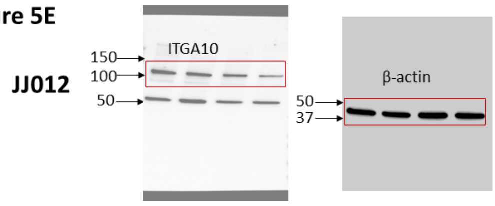

Sample	Parental	Mock	KO-1	KO-2
Density Ratio	0.13	0.12	0.04	0.01

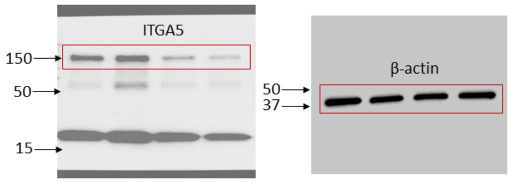

Sample	Parental	Mock	KO-1	KO-2
Density Ratio	0.7	1.17	0.25	0.09

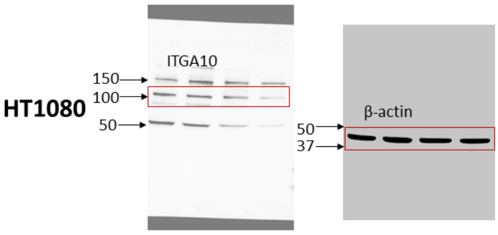

Sample	Parental	Mock	KO-1	KO-2
Density Ratio	0.88	0.72	0.44	0.03

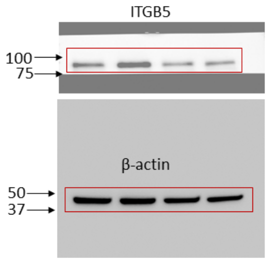

Sample	Parental	Mock	KO-1	KO-2
Density Ratio	0.57	1.01	0.31	0.35

Figure 5F

JJ012

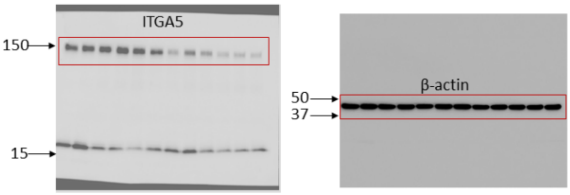

Sample	Parental	Parental	Parental	Mock	Mock	Mock	KO-1	KO-1	KO-1	KO-2	KO-2	KO-2
Density Ratio	0.69	0.73	0.86	0.92	0.87	0.64	0.23	0.51	0.3	0.15	0.18	0.13

HT1080

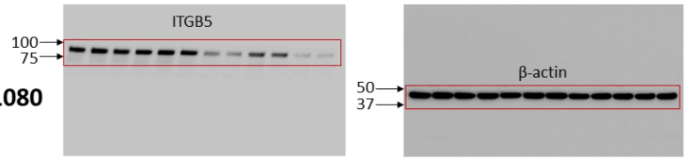

Sample	Parental	Parental	Parental	Mock	Mock	Mock	KO-1	KO-1	KO-1	KO-2	KO-2	KO-2
Density Ratio	0.71	0.8	0.75	0.74	0.95	0.74	0.25	0.22	0.47	0.40	0.09	0.08

Figure S2

JJ012 **HT1080**

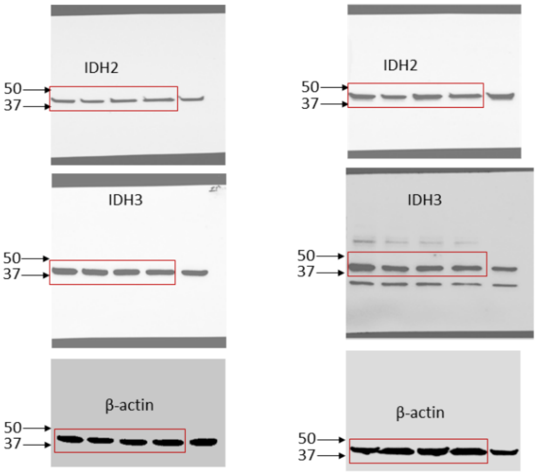

Sample	Parental	Mock	KO-1	KO-2
Density Ratio(IDH2)	0.82	0.79	0.90	0.88
Density Ratio(IDH3)	1.07	1.15	1.06	0.95

Sample	Parental	Mock	KO-1	KO-2
Density Ratio(IDH2)	0.99	0.61	0.88	0.74
Density Ratio(IDH3)	1.02	0.56	0.56	0.52

Figure S5

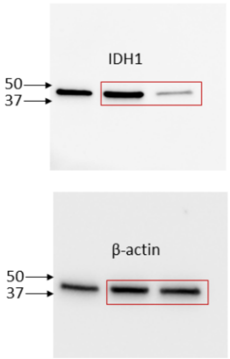

Sample	Parental	IDH1-KO
Density Ratio	1.08	0.2

Figure S6

JJ012

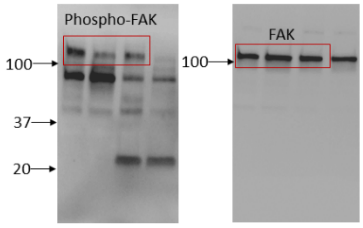

Sample	Parental	KO	KO (Octyl-D-2HG)
Density Ratio	1.17	0.41	0.68

HT1080

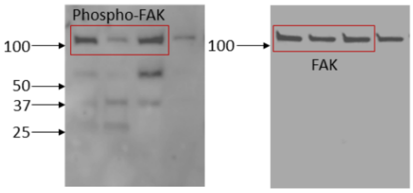

Sample	Parental	KO	KO (Octyl-D-2HG)
Density Ratio	0.85	0.40	1.2

Figure S9. blot images and density ratios from the main and supplementary figures. All the bands with molecular weight markers are shown. The red boxes indicate cropped regions presented in the actual figures. Density ratios were calculated with β-actin or total FAK density as protein references.

© 2020 by the authors. Licensee MDPI, Basel, Switzerland. This article is an open access article distributed under the terms and conditions of the Creative Commons Attribution (CC BY) license (http://creativecommons.org/licenses/by/4.0/).
